# First Report of an *Escherichia coli* Strain Carrying the Colistin Resistance Determinant *mcr-1* from a Dog in South Korea

**DOI:** 10.3390/antibiotics9110768

**Published:** 2020-11-02

**Authors:** Dong Chan Moon, Abraham Fikru Mechesso, Hee Young Kang, Su-Jeong Kim, Ji-Hyun Choi, Mi Hyun Kim, Hyun-Ju Song, Soon-Seek Yoon, Suk-Kyung Lim

**Affiliations:** Bacterial Disease Division, Animal and Plant Quarantine Agency, 177 Hyeksin 8-ro, Gimcheon-si, Gyeongsangbuk-do 39660, Korea; ansehdcks@korea.kr (D.C.M.); abrahamf@korea.kr (A.F.M.); kanghy7734@korea.kr (H.Y.K.); kimsujeong27@gmail.com (S.-J.K.); wlgus01@korea.kr (J.-H.C.); kimmh940301@naver.com (M.H.K.); shj0211@korea.kr (H.-J.S.); yoonss24@korea.kr (S.-S.Y.)

**Keywords:** colistin, dog, *E. coli*, *mcr*-*1*, One Health, plasmid

## Abstract

We studied the presence of the mobile colistin resistance gene *mcr*-*1* in *Escherichia coli* isolates recovered from fecal and urine samples of companion animals, that were collected from South Korea in 2018 and 2019. The *mcr*-*1* gene was detected in one colistin-resistant *E*. *coli* isolated from a diarrheic dog. The isolate exhibited additional resistance to multiple antimicrobials, including fluoroquinolones and third-generation cephalosporins. The *mcr*-*1* carrying isolate belonged to ST160. The pulsed-field gel electrophoresis pattern of our strain differed from those ST160 *E*. *coli* strains previously identified from chickens in Korea. The *mcr-1* gene was identified in the IncI2 plasmid. It was also transferred to *E. coli* J53 recipient strain, with a conjugation efficiency of 2.8 × 10^−4^. Average nucleotide identity analysis demonstrated that the *mcr*-*1*-carrying plasmid in this study was closely related to those from patients in Korea. To the best of our knowledge, this is the first report of *mcr*-1 carrying *E. coli* from a companion animal in South Korea. Our findings support One Health approach is necessary to prevent the dissemination of this high-risk gene.

## 1. Introduction

Colistin is the last-resort antibiotic against multidrug-resistant Gram-negative bacteria. Since the first report of *mcr*-*1* gene in China in 2016 [1], 10 *mcr* genes have been described so far i.e., *mcr-1* to *mcr-10* [2]. The *mcr* genes code for phosphoethanolamine transferases which catalyze the attachment of phosphoethanolamine to lipopolysaccharides-lipid A in the outer membrane of Gram-negative bacteria. This leads to a reduction of the negative charge of lipopolysaccharides upon structural alteration of lipid A and confers resistance to colistin [3]. Horizontal transfer of the *mcr* genes contributes to the rapid spread of colistin resistance among *Enterobacteriaceae* [4].

*Escherichia coli* plays an important role in colistin resistance because it is the main *mcr* gene carrier [4] and can be easily transferred among different animal hosts [5]. Plasmid-mediated mobile colistin resistance gene *mcr*-*1* was observed in *E. coli* from humans [1,6,7], food [1,8] and companion animals [9,10,11] in other countries. In the Republic of Korea (Korea), after our first report of *mcr*-*1*-carrying *E. coli* in livestock [12], the *mcr*-*1* gene was reported in *E. coli* isolated from humans, food animals, and fresh vegetables [13,14,15]. Despite the close interaction between companion animals and humans, no attempt has been made to identify *mcr*-*1*-carrying *E. coli* from companion animals in Korea to date. Here, we report the first detection of *mcr*-*1* in colistin-resistant *E. coli* isolated from a dog.

## 2. Materials and Methods

### 2.1. Identification of Colistin-Resistant E. coli

*E. coli* isolates were recovered from fecal (diarrheic and non-diarrheic) and urine samples of dogs (1374) and cats (441) from seven metropolitan cities in Korea in 2018 and 2019 (Table S1). Isolation and identification of *E. coli* isolates were performed using Eosin methylene blue agar (EMB, Becton Dickinson, Sparks, Baltimore, MD, USA) and MacConkey agar plates (MAC, BD, Spark, Baltimore, MD, USA). Isolates were then confirmed by matrix-assisted laser desorption ionization‒time-of-flight mass spectrometry (MALDI-TOF, Biomerieux, Marcy L’Etoile, France). Only a single isolate per sample was considered for susceptibility study. The minimum inhibitory concentration (MIC) of colistin was determined by the broth microdilution method [16] in KRNV5F Sensititre Panel following the manufacturer’s instruction (Trek Diagnostic Systems, Cleveland, OH, USA). The MIC values were interpreted according to the EUCAST breakpoint (>2 µg/mL) [17]. Additionally, PCR amplification was performed to investigate the *mcr-1* gene carriage of isolates demonstrating colistin resistance using primer pairs and PCR conditions described previously [1].

### 2.2. Conjugation Assay

Conjugation was performed by the filter mating method at 37 °C using azide-resistant *E. coli* J53 as the recipient strain and colistin-resistant isolates as donor strains. The experiments were conducted in triplicate onto Luria–Bertani agar plate, using a 1:10 donor to recipient ratio. Transconjugant bacteria were selected on Mueller-Hinton agar plates containing sodium azide (100 mg/mL) and colistin (1 μg/mL) [18]. Transfer frequencies were calculated based on the number of transconjugants obtained per donor. The transconjugant was confirmed by PCR detection of the *mcr-1* gene as described above.

### 2.3. Molecular Characterization of mcr-1 Carrying E. coli

Pulsed-field gel electrophoresis (PFGE) of *mcr-1* positive isolates was conducted using genomic DNA prepared in agarose blocks, digested with Xbal enzyme (TaKaRa, Shiga, Japan), as described previously [19]. The banding profiles were analyzed using Bionumerics software and the genetic relatedness of the isolates was calculated using the unweighted pair-group method. Additionally, molecular typing of *mcr-1* carrying isolates was carried out according to the protocols specified at the *E. coli* multilocus sequence typing website [20].

### 2.4. Comparative Analysis of mcr-1 Carrying Plasmids

The *mcr-1* carrying plasmid pK19EC149 was sequenced (Pacific Biosciences, Menlo Park, CA, USA; http://pacificbiosciences.com/) (GenBank accession number. CP050290) and compared with those of previously reported strains (Table 1). Briefly, nucleotide sequences of *mcr-1* carrying plasmids were downloaded from the GenBank nucleotide database. As the sequences have different starting points, sequences were rotated for accurate alignment so that start sites of sequences were set as RepA using GAMOLA2 [21]. Average Nucleotide Identity (ANI) values were calculated with pairwise genome alignment of sequences by using the ANI-blast method implemented in PYANI (v.0.2.9) [22] and the phylogenetic tree is reconstructed based on the ANI values. The sequence of each plasmid was aligned using Blastn (v 2.8.1) [23] and compared using EasyFig (v.2.2.3) [24].

## 3. Results and Discussion

Overall, 1202 *E. coli* isolates (288 cat and 914 dog isolates) were identified from fecal and urine samples of dogs and cats. Susceptibility testing demonstrated two colistin-resistant isolates from diarrheic dogs in Seoul city (MIC = 8 µg/mL). PCR and sequencing analysis identified the *mcr-1* gene in one of the colistin-resistant isolates. The *mcr-1* prevalence in *E. coli* isolates in this study was lower than those previously reported in *E. coli* recovered from companion animals in Argentina (1.9%) [25], Beijing, China (2.3%) [27], and Guangzhou, China (6.25%) [28]. However, it was almost similar to our previously described *mcr-1* detection in food animals in Korea [12]. This is the first report of *mcr-1*-carrying *E. coli* from a dog in Korea. Considering the close relationship between humans and dogs, the observation of *mcr-1*-carrying *E. coli* in dog feces is a potential risk to public health.

**Figure 1:**
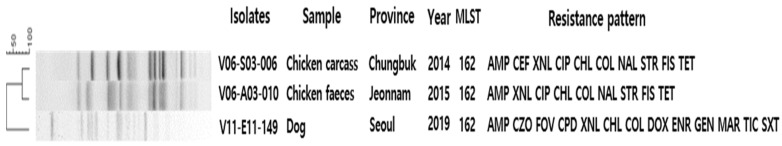
*Xbal*-digested pulsed-field gel electrophoresis patterns of *mcr*-*1*-carrying *E. coli* strains isolated from a diarrheic dog and chickens in Korea. AMP, ampicillin; CPD, cefpodoxime; CEF, cephalothin; CHL; chloramphenicol; CIP, ciprofloxacin; COL, colistin; CZO, cefazolin; DOX, doxycycline; ENR, enrofloxacin; FIS, sulfisoxazole; FOV, cefovecin; GEN, gentamicin; MAR, marbofloxacin; NAL, nalidixic acid; STR, streptomycin; SXT, trimethoprim/sulfamethoxazole; TET, tetracycline; TIC, ticarcillin; XNL, ceftiofur.

The *mcr-1*-carrying strain belonged to ST162 and commensal subgroup B1, which concurred with our previous finding in chickens [12]. However, the PFGE pattern of our *mcr-1*-carrying ST162 *E. coli* strain differed from those of chicken strains (Figure 1) [12,15]. Zhang et al. [29] identified ST162 *E. coli* harboring multiple antibiotic-resistant genes, including *mcr-1* from a dog in China. ST162 *E. coli* that belonged to subgroup B1 possessed high virulence and was linked with extraintestinal pathogenic *E. coli*-associated human infections [30]. The *mcr-1*-carrying *E. coli* exhibited additional resistance to multiple antimicrobials, including fluoroquinolones and third-generation cephalosporins (Figure 1). While, the other colistin-resistant isolate (*mcr-1*-negative isolate) was susceptible to all of the tested antimicrobials including aminoglycosides, cephalosporins, and fluoroquinolones. The broth mating assay indicated that the strain was capable of transferring its gene to the recipient *E. coli* J53 strain. Indeed, the conjugation efficiency of our isolate (2.8 × 10^−4^) was at least ten times higher than those of previous isolates from humans in Korea [15]. Nonetheless, resistance determinants other than colistin were not observed in the transconjugant.

**Figure 2:**
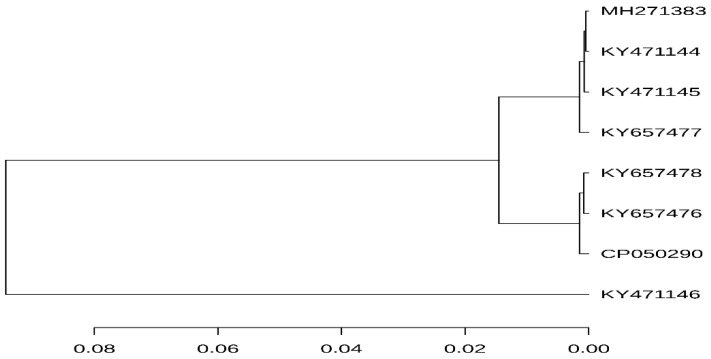
ANI analysis was performed using the ANI-blast method implemented in PYANI (v.0.2.9) and the tree was generated based on the ANI values. The horizontal lines represent the 95% threshold value. The scale bar represents sequence divergence. Detailed information on the plasmids source is summarized in Table 1.

**Figure 3:**
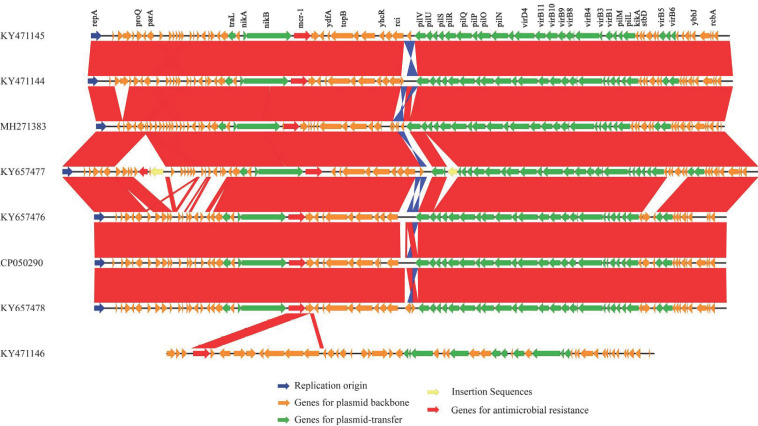
Comparative analyses of the *mcr-1*-carrying plasmids from *E. coli* strains isolated from food animals, humans, and a dog. The sequence of each plasmid (Table 1) was aligned using Blastn (v 2.8.1) and compared using EasyFig (v.2.2.3). Highly conserved regions (>96% identity value) with normal alignment are indicated in red, and regions with inverted alignment are indicated in blue.

According to the 95% ANI threshold, the *mcr-1*-carrying plasmid pK19EC149 from this study was closely related to those from patients in Korea (GenBank accession no. KY657476 and KY657478) (Figure 2). Additionally, the *mcr-1* carrying plasmid IncI2 pK19EC149 (60,864 bp) had a similar size and highly conserved backbone (>96%) to other IncI2 plasmids detected in *E. coli* strains from humans (KY657476 and KY657478) and chickens (KY471144, KY471145) in Korea (Figure 3). The findings indicate that the plasmids might have evolved from a single ancestor, or one might have evolved from the other. The IncI2 plasmids, which have a broad host range, are commonly associated with the acquisition and dissemination of new antibiotic-resistant genes. As well, they are known to adapt to new bacterial hosts [31,32].

Colistin is not commonly used to treat companion animals in Korea; however, it is widely used in the livestock industry. Additionally, *mcr-1* carrying *E. coli* was mainly identified in food animals in the country [12,14]. Thus, food animals are considered responsible for spreading the *mcr-1* gene. Nonetheless, we isolated the *mcr-1* carrying *E. coli* from a dog in the urban areas of the Seoul metropolitan. This implies that the dog had minimal or no contact with food animals. Considering the close and frequent contact between humans and companion animals, our results might suggest that *mcr-1*-carrying *E. coli* could be transferred between dogs and humans. Consistent with this study, the transmission of *mcr*-carrying *E. coli* between humans and companion animals, especially dogs, was reported in China [9,10]. Additionally, *E. coli* harboring the *mcr-1* gene has been frequently identified from environmental samples [33,34,35]. Guenther et al. [36] showed a clear link between *mcr-1*-carrying *E. coli* isolated from the environment and those from humans and dogs. Therefore, the *mcr-1*-carrying *E. coli* in this study might also originate from the natural environment.

In conclusion, dogs can serve as a reservoir of *mcr-1* carrying *E. coli*, adding another layer of intricacy to the rapidly evolving epidemiology of plasmid-mediated colistin resistance in the community. Thus, a “One Health” based strategy and a detailed knowledge of antimicrobial usage in humans and companion animals are needed to reduce the dissemination of colistin-resistant *E. coli*.

## Figures and Tables

**Table 1:** Lists of plasmids identified in isolates recovered from humans, dogs, and food animals.

Plasmids	GenBank Accession Number	Bacterial Species	Host	Country	Reference
pV80	MH271383	*E. coli*	Dog	Argentina	[25]
pEC006	KY471144	*E. coli*	Chicken	Korea	[26]
pEC019	KY471145	*E. coli*	Chicken	Korea	[26]
pEC111	KY471146	*E. coli*	Pig	Korea	[26]
pCREC-527-4	KY657476	*E. coli*	Human	Korea	[15]
pCRENT-301-1	KY657477	*Enterobacter aerogenes*	Human	Korea	[15]
pUSU-ECO-12704-4	KY657478	*E. coli*	Human	Korea	[15]
pK19EC149	CP050290	*E. coli*	Dog	Korea	This study

## Supplementary Materials

The following are available online at https://www.mdpi.com/2079-6382/9/11/768/s1. Table S1: The number of fecal samples, urine samples, and *E. coli* isolates collected from dogs and cats in 2018 and 2019 in Korea.
