# Supplementary material for: First Report of an Escherichia coli Strain Carrying the Colistin Resistance Determinant mcr-1 from a Dog in South Korea
Source: Antibiotics (Basel). 2020 Nov 2;9(11):768. doi: 10.3390/antibiotics9110768 (PMC7694106; doi:10.3390/antibiotics9110768)
Supplement: Supplementary file 1 [file antibiotics-09-00768-s001.pdf]

antibiotics

MDPI

Table S1. The number of fecal samples, urine samples, and *E. coli* isolates collected from dogs and cats in 2018 and 2019 in Korea.

Sample type	Source	2018			2019		
		Dogs	Cats	Total	Dogs	Cats	Total
Faeces (non-diarrheic)	No. of hospitals	27	18	45	27	26	53
	No. of animals	133	56	189	217	75	292
	No. of samples	133	56	189	217	75	292
	No. of isolates	98	44	142	155	59	214
Faeces (diarrheic)	No. of hospitals	38	30	68	47	33	80
	No. of animals	277	111	388	577	135	712
	No. of samples	277	111	388	581	137	718
	No. of isolates	196	72	268	414	103	517
Urine	No. of hospitals	26	15	41	27	16	43
	No. of animals	89	30	119	81	34	115
	No. of samples	90	30	120	81	34	115
	No. of isolates	26	3	29	25	7	32
